# Echinacoside Suppresses Amyloidogenesis and Modulates F-actin Remodeling by Targeting the ER Stress Sensor PERK in a Mouse Model of Alzheimer’s Disease

**DOI:** 10.3389/fcell.2020.593659

**Published:** 2020-11-19

**Authors:** Yuan Dai, Guanghui Han, Shijun Xu, Yongna Yuan, Chunyan Zhao, Tao Ma

**Affiliations:** ^1^Dongfang Hospital, Beijing University of Chinese Medicine, Beijing, China; ^2^Institute of Material Medica Integration and Transformation for Brain Disorders, Chengdu University of Traditional Chinese Medicine, Chengdu, China; ^3^School of Health Preservation and Rehabilitation, Chengdu University of Traditional Chinese Medicine, Chengdu, China; ^4^School of Pharmacy, Chengdu University of Traditional Chinese Medicine, Chengdu, China; ^5^School of Information Science and Engineering, Lanzhou University, Lanzhou, China; ^6^School of Pharmacy, Lanzhou University, Lanzhou, China

**Keywords:** Alzheimer’s disease, echinacoside, endoplasmic reticulum stress, filamin-A, amyloid β, f-actin, PERK, eIF2α

## Abstract

Endoplasmic reticulum stress (ERS) plays a vital and pathogenic role in the onset and progression of Alzheimer’s disease (AD). Phosphorylation of PKR-like endoplasmic reticulum kinase (PERK) induced by ERS depresses the interaction between actin-binding protein filamin-A (FLNA) and PERK, which promotes F-actin accumulation and reduces ER-plasma membrane (PM) communication. Echinacoside (ECH), a pharmacologically active component purified from *Cistanche tubulosa*, exhibits multiple neuroprotective activities, but the effects of ECH on ERS and F-actin remodeling remain elusive. Here, we found ECH could inhibit the phosphorylation of PERK. Firstly ECH can promote PERK-FLNA combination and modulate F-actin remodeling. Secondly, ECH dramatically decreased cerebral Aβ production and accumulation by inhibiting the translation of BACE1, and significantly ameliorated memory impairment in 2 × Tg-AD mice. Furthermore, ECH exhibited high affinity to either mouse PERK or human PERK. These findings provide novel insights into the neuroprotective actions of ECH against AD, indicating that ECH is a potential therapeutic agent for halting and preventing the progression of AD.

## Introduction

Alzheimer’s disease (AD) is the most prevalent cause of dementia in elderly people and is symptomatically characterized by progressive, age-dependent impairment in memory, cognitive function, and behavior (Reitz et al., 2011). Brains of AD patients can be pathologically identified by the progressive accumulation of insoluble amyloid plaques composed of neurotoxic amyloid β protein (Aβ), which is believed to play a central pathogenic role in this devastating illness (Masters and Selkoe, 2012). Aβ is proteolytically derived from the larger amyloid precursor protein (APP) by two proteolytic enzymes, β-secretase (beta-site APP cleaving enzyme 1, BACE1) and γ-secretase. BACE1 is the rate-limiting enzyme that catalyzes the initial cleavage of APP and gives rise to Aβ. The level of BACE1 is expected to play a fundamental role in the aetiopathogenesis of AD. Emerging evidence has shown that the level of BACE1 protein is aberrantly increased in the brains of AD patients as well as in different AD transgenic mouse models, whereas the level of BACE1 mRNA tends to be constant (Holsinger et al., 2002; Preece et al., 2003; Yang et al., 2003; Li et al., 2004; O’connor et al., 2008; Kim et al., 2018). This observation indicates that modulation of the translation of BACE1 will be a promising target for designing therapeutic agents to prevent or treat this neurodegenerative disorder (Ohno, 2006; Vassar and Kandalepas, 2011; Yan and Vassar, 2014).

The endoplasmic reticulum (ER) is an important apparatus that contributes to both protein modification and processing. Accumulated misfolded or aggregated proteins, such as Aβ, in the ER can trigger endoplasmic reticulum stress (ERS) and lead to many protein-folding diseases, including AD (Ron and Walter, 2007; Rozpedek et al., 2015; Bell et al., 2016; Hughes and Mallucci, 2018). ERS can activate a set of pro-survival signaling pathways termed the unfolded protein response (UPR). The UPR rapidly depresses global protein synthesis to deal with the accumulation of unfolded proteins, providing a protective mechanism capable of restoring proteostasis (Rozpedek et al., 2015; Hughes and Mallucci, 2018). During the UPR, eukaryotic initiation factor-2α (eIF2α), a key translational initiator, is activated via phosphorylation, leading to a halt in general translation and translational activation of a subset of mRNAs (Chang et al., 2002; Ferrer, 2002; Ma et al., 2013; Devi and Ohno, 2014). Sustained eIF2α phosphorylation and subsequent persistent repression of global protein synthesis, which result in memory impairments and neurodegeneration during chronic diseases such as AD, are observed in the brains of sporadic AD patients as well as in different AD transgenic mouse models (Chang et al., 2002; Ferrer, 2002; Kim et al., 2007; O’connor et al., 2008; Morel et al., 2009; Ohno, 2014). Interestingly, although the phosphorylation of eIF2α at Ser51 inhibits general translation initiation, it paradoxically activates translation of BACE1. Consistent with the abnormally persistent hyperphosphorylation of eIF2α, the expression of BACE1 is markedly elevated in AD brain, leading to deficits in neuronal plasticity and memory formation (Ma et al., 2013; Devi and Ohno, 2014).

The phosphorylation of eIF2α is controlled by four protein kinases, general-control non-derepressible-2 kinase (GCN2), double-stranded RNA-activated protein kinase (PKR), haeme-regulated inhibitor kinase (HRI), and PKR-like endoplasmic reticulum kinase (PERK) (Ohno, 2014). Among these four kinases, PERK is associated with the UPR and is the major kinase that activates eIF2α in the brain, which is overly activated via phosphorylation in the AD brain (O’connor et al., 2008; Ohno, 2014). PERK/eIF2α signaling dysfunction is a common mechanism leading to neurodegenerative diseases, including AD (Hetz and Mollereau, 2014; Bell et al., 2016). Since dysregulation of the PERK/eIF2α pathway is a potential pathophysiologic factor contributing to AD, PERK has emerged as a novel potential therapeutic target for AD treatment (Ma and Klann, 2014; Rozpedek et al., 2015; Ohno, 2018). Diminishing eIF2α phosphorylation via fore-brain-specific conditional deletion, genetic haploinsufficiency, or pharmacological manipulation of PERK mitigates cerebral Aβ accumulation by reducing BACE1 levels and ameliorates cognition deficits in AD transgenic mice (Ma et al., 2013; Zhu et al., 2013b; Devi and Ohno, 2014).

During the ERS, the loss of ER Ca^2+^ homeostasis could accelerate the formation of ER-plasma membrane (PM) communication. As a stress response to ERS, one of the important biological functions of ER-PM contact site is to restore the homeostasis of Ca^2+^ in cytoplasm and ER. Van Vliet et al. (2017) confirmed that dimerized PERK interacted with filamin-A (FLNA) which is a key F-actin modulation protein. Furthermore, PERK-FLNA interaction drives the expansion of ER-PM juxtapositions by regulating F-actin network remodeling. Notably, sustained phosphorylation of PERK would depress PERK-FLNA combination, thereby reducing the ER-PM contact sites, leading to further deterioration of ERS.

**FIGURE 1:**
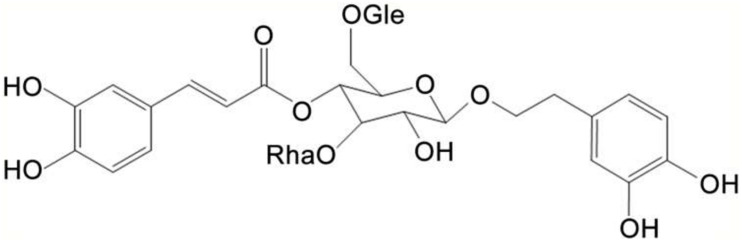
Chemical structure of Echinacoside (ECH).

Echinacoside (ECH) (Figure 1) is one of the major phenylethanoid glycosides isolated and purified from *Cistanche tubulosa*, a parasitic plant native to northwestern China, which is used as a traditional Chinese herbal medicine with anti-senility and antifatigue effects (He et al., 2009). Consisting of a phenylpropanoid and a phenylethanoid glycosidically linked to a trisaccharide moiety, ECH is a hydrophilic polyphenol glycoside, which has a strong activity of scavenging superoxide anion, hydroxyl radical, and lipid radicals (Li et al., 1992) and can inhibit the autoxidation of linoleic acid (Zheng et al., 1993). Containing caffeoyl and hydroxyphenylethyl moieties, ECH exhibits a wide range of bioactivities such as free radical scavenging, antioxidant and anti-inflammatory effects (Facino et al., 1995; Heilmann et al., 2000; Sloley et al., 2001; Pellati et al., 2004; Dalby-Brown et al., 2005).

Many studies have suggested that ECH exhibits strong neuroprotective effects. ECH elicits neuroprotection against neuronal injury and apoptosis induced by 1-methyl-4-phenyl-1,2,3,6-tetrahydropyridine (MPTP), 6-hydroxydopamine, rotenone, hydrogen peroxide (H_2_O_2_), tumor necrosis factor-α (TNFα), and ultraviolet B (UVB) irradiation *in vitro* and *in vivo* (Geng et al., 2007; Kuang et al., 2009, 2010; Zhu et al., 2012, 2013a; Wang et al., 2015). Furthermore, ECH can ameliorate the memory impairment in senescence-accelerated prone inbred strains (SAMP) of mice and rats with bilateral middle carotid artery occlusion (MCAO) (He et al., 2009; Liu et al., 2013). Thus, many researchers suggest that ECH is a potential therapeutic natural compound for AD, Parkinson’s disease (PD) and vascular dementia (VD) (Chen et al., 2007; Zhao et al., 2010; Zhu et al., 2012, 2013a; Liu et al., 2013). ECH and *Cistanche tubulosa* extract, with ECH as the main component, ameliorate the cognition deficits and Aβ deposition and reverse the cortical cholinergic dysfunction caused by Aβ42 in a rat model of Alzheimer’s disease (Wu et al., 2014; Shiao et al., 2017). However, the possible therapeutic target of ECH in preventing AD deterioration involving ERS and F-actin network remodeling has not yet been ascertained.

Different AD mouse models and PERK gene-targeting approaches consistently suggest that overactivation of the PERK-dependent eIF2α phosphorylation pathway may cause memory deficits associated with AD (Ma et al., 2013; Devi and Ohno, 2014). The present study was designed to investigate the disease-modifying effects of ECH on cognitive impairment, Aβ accumulation, and the associated ERS, as well as the underlying mechanisms in APPswe/PS1dE9 double-transgenic mice that develop AD-like symptoms and act as a model of familial AD (Savonenko et al., 2005; Zhou et al., 2015).

## Materials and Methods

### Animals

Three-month-old male APPswe/PS1dE9 (2 × Tg-AD) mice, harboring human APPswe (Swedish mutations K594N/M595L) and presenilin-1 with exon 9 deleted (PS1dE9) under the control of the constitutively active CMV promoter (Savonenko et al., 2005; Garcia-Alloza et al., 2006; Zhou et al., 2015), with a pre-existing subset of behavioral and pathological features of AD, were used to more closely mimic the clinical setting of AD. The non-transgenic (Non-Tg) littermates were used as a control. All mice were purchased from the Beijing HFK Bioscience Co., LTD (Beijing, China), and were adaptively reared for 30 days in the SPF animal laboratory in Dongfang Hospital, Beijing University of Chinese Medicine, China.

After adaptive feeding, 4-month-old 2 × Tg-AD mice and Non-Tg mice were randomly separated into 4 groups: Vehicle (normal saline, NS) + Non-Tg (*n* = 20), Vehicle (normal saline, NS) + 2 × Tg-AD (*n* = 20), ECH + Non-Tg (*n* = 20) and ECH + 2 × Tg-AD (*n* = 20). All mice were individually caged at an ambient temperature of 23 ± 1°C and relative humidity 55 ± 5% under a 12:12 h light/dark cycle and received food and water *ad libitum*. The animals were handled according to the NIH Guide for the Care and Use of Laboratory Animals (NIH Publications No. 80-23, revised 1996). All animal studies were approved by the Animal Care & Welfare Committee of Dongfang Hospital, Beijing University of Chinese Medicine, China.

### Administration of Drugs

Echinacoside (purity > 98%, Figure 1) was provided by Xi’an Haoxuan Bio-Tech (Xi’an, China) and dissolved in NS. ECH-treated mice were received 30 mg/kg b.w. ECH daily i.g. for 180 days and vehicle-treated mice received NS of the same volume as ECH daily i.g. for 180 days.

### Morris Water Maze Test

As described by Lv et al. (2015), the Morris water maze test was performed to evaluate the spatial memory in response to treatment with ECH in 2 × Tg-AD mice. A maze of 120 cm in diameter and 35 cm in height was filled with water at 23 °C to a depth of 25 cm. The escape platform (8 cm diameter), which was placed at a fixed position in the center of one quadrant, was 1 cm below the water surface during the place navigation test and 1.5 cm above the water surface during the visible-platform test. The room contained several fixed visual cues on the wall. The day before testing, each mouse was individually placed into the center of the pool and allowed to swim for 60 s to familiarize themselves with the environment of the water maze. The mice were then trained to climb onto the platform from the water as a means of escape.

#### Place Navigation Test

The place navigation test consisted of 7 training days (Days 1–7) and two trials per day with a 1 h inter-trial interval. A quadrant was selected randomly, and the mice were placed into the water along the wall with their back against the platform. The mice were allowed 60 s to escape the water by locating the hidden platform. The escape latency was defined as the length of time that the mice required to reach the platform. If a mouse failed to find the platform within 60 s, it was placed on the platform for 15 s before the start of the next trial, and the latency was recorded as 60 s. All experiments were conducted at approximately the same time each day. The investigator was blinded to mouse genotypes until all behavioral tests were completed.

#### Spatial Probe Test

The spatial probe test was performed 24 h after the final day of the place navigation test (Day 8) to assess the spatial memory. The platform was removed from the maze, and mice were allowed to swim freely for 60 s. The swimming trajectory, the number of crossings of the area of the removed platform, the time spent in the target quadrant where the platform was previously located, and the time spent in the opposite quadrant were recorded to evaluate the memory capacity of mice.

#### Visible-Platform Test

During Days 9–12, the visible-platform test was performed to evaluate the difference in visual-motor abilities or motivation among the experimental groups. For the visible-platform test, the platform was raised above the water surface and placed in a different position from the place navigation test. Mice were given four trials per day with 30 min inter-trial intervals.

### Immunohistochemistry and Aβ Plaque Load Quantification

After the Morris water maze tests, animals were anesthetized with chloral hydrate and perfused transcardially with NS, followed by ice-cold 4% paraformaldehyde in NS. Brains were removed and fixed in 4% paraformaldehyde and paraffin-enclosed for examination. Serial 4-μm coronal paraffin-embedded sections were used for immunohistochemistry, and the VECTASTAIN Elite ABC Universal PLUS kit (Vector Laboratories, Burlingame, CA, United States) was used to determine the distribution of Aβ42-positive plaques in the mouse brains. Briefly, paraffin sections were deparaffinized and rehydrated, washed in distilled water, and then subjected to heat-mediated antigen retrieval treatment. Endogenous peroxidase activity was eliminated by incubation in blocking solution for 10 m and then washing in phosphate buffer solution (PBS) for 5 m. The sections were blocked for 1 h with 2.5% normal horse serum at 37°C and incubated overnight with a monoclonal Aβ antibody (6E10) (1:200, 803015, BioLegend, San Diego, CA, United States) at 4°C in a humidified chamber. Then, the sections were washed in PBS and incubated with the biotinylated horse anti-mouse IgG secondary antibody for 1 h. After washing with PBS, the sections were incubated with the VECTASTAIN Elite ABC Reagent for 30 min and visualized by Mix ImmPACT DAB EqV solution. The sections were then counterstained by hematoxylin.

For the quantification of Aβ42-positive plaque load, digital images were captured with an Olympus IX71 microscope using a single-exposure setting as follows: Non-Tg Veh, *n* = 5; Non-Tg ECH, *n* = 6; 2 × Tg-AD Veh, *n* = 5; 2 × Tg-AD ECH, *n* = 5; and 6 sections through the hippocampus or cortex formation per mouse were calculated. Plaque load was defined as the % area, i.e., the positive area/total area × 100%, and semiquantitatively analyzed by ImageJ 1.46r (NIH, United States). The data are presented as mean ± SEM.

### Aβ ELISA

The levels of cerebral total Aβ, Aβ 40, and Aβ 42 were determined by using the Aβ1-x ELISA kit (Immuno-Biological Laboratories, Gunma, Japan) and Invitrogen mouse Aβ40 and Aβ42 ELISA kits (Thermo Fisher Scientific, Camarillo, CA, United States), according to the manufacturers’ instructions. After behavioral testing, the animals were anesthetized with chloral hydrate and sacrificed via decapitation. The brains of the vehicle- and ECH-treated mice were immediately dissected and homogenized in ice-cold RIPA lysis buffer [50 mM Tris-HCl [pH 7.4], 150 mM NaCl, 1% NP-40, 0.1% sodium dodecyl sulfate (SDS)] (Applygen Technologies, Beijing, China) supplemented with cOmplete protease inhibitor cocktail and PhosSTOP phosphatase inhibitor cocktail (Roche Diagnostics GmbH, Mannheim, Germany) with a Dounce homogenizer on ice. Brain homogenates were centrifuged at 12,000 *g* and 4°C for 10 min. One milliliter of the supernatant was set aside and used for Western blot. The remaining lysate was subjected to ELISA. Sample protein concentration was quantitated by the Pierce BCA protein assay kit (Thermo Fisher Scientific, Rockford, IL, United States). The final Aβ values were normalized to total protein levels (*n* = 6 per group).

### RNA Isolation and Quantitative PCR

Total RNA was isolated using the RNeasy Mini kit (Qiagen, Valencia, CA, United States) according to the manufacturer’s instructions. RNA concentration and purity, from the absorbance at 260/280 nm, were analyzed using a BioPhotometer plus (Eppendorf, Hamburg, Germany). For quantitative reverse transcription-polymerase chain reaction (qRT-PCR) analysis, 1.5 μg total RNA from each sample was used for first-strand cDNA synthesis and qPCR using the Invitrogen EXPRESS One-step SYBR^TM^ GreenER^TM^ Kit (Thermo Fisher Scientific, Camarillo, CA, United States). qRT-PCR was conducted using a 7300 sequence analyzer (Applied Biosystems, Foster City, CA, United States); data were analyzed using Applied Biosystems SDS 1.2. The custom probes for mouse BACE1 were forward, 5′-GATGGTGGACAACCTGAG-3′, and reverse: 5′-CTGGTAGTAGCGATGCAG-3′. BACE1 mRNA levels for each experimental group were normalized against 18s rRNA and quantified using the comparative C_*T*_ method.

### Antibodies and Western Blot Analysis

The following antibodies were used in Western blot analysis: polyclonal anti-BACE1 antibody (1:1000, 195111, Calbiochem, Merck Millipore, Darmstadt, Germany), monoclonal anti-sAPPβsw (6A1) antibody (1:500, 10321, Immuno-Biological Laboratories, Gunma, Japan), Chemicon monoclonal anti-full-length-APP (flAPP) (22C11) antibody (1:1000, #MAB348, Merck Millipore, Darmstadt, Germany), polyclonal anti-A-disintegrin and metalloproteinase 10 (ADAM10) antibody (1:1000, AB19026, Merck Millipore, Darmstadt, Germany), polyclonal anti-presenilin 1 (PS1) antibody (1:500, #3622, Cell Signaling Technology, Danvers, MA, United States), monoclonal anti-GRP78 antibody (1:2000, 610978, BD Biosciences, San Jose, CA, United States), polyclonal anti-eIF2α antibody (1:1000, #9722, Cell Signaling Technology, Danvers, MA, United States), monoclonal anti-phospho-eIF2α (Ser51) (D9G8) antibody (1:1000, #3398, Cell Signaling Technology, Danvers, MA, United States), monoclonal anti-PERK (C33E10) antibody (1:1000, #3192, Cell Signaling Technology, Danvers, MA, United States), polyclonal anti-phospho-PERK antibody (1:500, 649401, BioLegend, San Diego, CA, United States), monoclonal anti-β-actin antibody (C4) (1:2000, sc-47778, Santa Cruz, Dallas, TX, United States), goat anti-mouse IgG-HRP (sc-2302) and goat anti-rabbit IgG-HRP (sc-2004) secondary antibodies (1:5000, Santa Cruz, Dallas, TX, United States). The protein samples were mixed with the loading buffer containing 50 mM Tris-HCl (pH 6.8), 10% glycerol (V/V), 20% SDS (g/mL), 100 mM DTT and 0.1% bromophenol blue (g/mL) and heated at 95°C for 10 min. Then, 10% SDS-poly-acrylamide gel electrophoresis (PAGE) was performed using a Mini-PROTEAN system (Bio-Rad, Hercules, CA, United States) and SeeBlue Plus2 pre-stained protein standard (Life Technologies, Carlsbad, CA, United States). Each lane was loaded with 50 μg of protein. After electrophoresis, the proteins were transferred to Immobilon-P polyvinylidene difluoride (PVDF) membranes (Millipore, Billerica, MA, United States) at 295 mA for 1.5 h. Non-specific binding was blocked with 50 g/L Difco^TM^ skim milk (BD Bioscience, Franklin Lakes, NJ, United States) in TBST (50 mM Tris-HCl, pH 7.4, 200 mM NaCl, 0.5 mM Tween-20). The blots were incubated overnight at 4 °C with different primary antibodies, and β-actin served as a loading control. Then, the membranes were washed with TBST 4 times and incubated with the secondary antibodies conjugated to peroxidase for 1 h at room temperature. The bands were detected with the Pierce ECL Western Blotting Substrate (Thermo Fisher Scientific, Rockford, IL, United States) using a GeneGnome XRQ bioimaging system (Syngene, Cambridge, United Kingdom). Bands were quantified using the software ImageJ 1.46r (NIH, United States).

### BACE1 (β-Secretase) Activity Assay

Enzymatic activity of BACE1 (β-secretase) was determined using the β-secretase activity fluorometric assay kit (Biovision, Milpitas, CA, United States) according to the manufacturer’s instructions. Briefly, brain tissues were mixed with 2 volumes of ice-cold Extraction Buffer and homogenized on ice. After 10 min incubation on ice, the extracts were centrifuged at 10,000 × *g* for 5 m. Then, 50 μL supernatant was mixed with an equal volume of 2× reaction buffer and 2 μL substrate. The plates were kept in the dark at 37°C for 1 h, and the fluorescence was recorded at Synergy H1 Hybrid Multi-Mode Microplate Reader (BioTek, Winooski, VT, United States) with Ex. 340 nm and Em. 500 nm. BACE1 activity was expressed as relative fluorescence units per micrograms of protein sample.

### α-Secretase Activity Assay

Enzymatic activity of α-secretase was measured using a commercial kit (AnaSpec, Fremont, CA, United States) according to the operation manual. Briefly, brain tissues were homogenized in ice-cold assay buffer and incubated on ice for 15 min. The extracts were centrifuged for 15 min at 10,000 × *g*. Then, 50 μL supernatant was mixed with an equal volume of the α-secretase substrate. The plate was incubated at 37°C in the dark for 1 h, and the fluorescence intensity at Ex/Em = 490 nm/520 nm was recorded with Synergy H1 Hybrid Multi-Mode Microplate Reader. the α-Secretase activity was expressed as relative fluorescence units per microgram of protein sample.

### γ-Secretase Activity Assay

The γ-secretase activity was measured with a commercial kit (R&D Systems, Wiesbaden, Germany) according to the manufacturer’s protocol. Briefly, 50 μL brain tissue lysate was mixed with an equal volume of 2× reaction buffer and 5 μL of the substrate. The reaction mixture was incubated in the dark at 37°C for 1.5 h. The fluorescence was recorded at Synergy H1 Hybrid Multi-Mode Microplate Reader with Ex. 340 nm and Em. 500 nm. γ-Secretase activity was expressed as relative fluorescence units per microgram of protein sample.

### Transmission Electron Microscopy

The mice of each group were anesthetized and perfused with cold 0.9% NS and 4% paraformaldehyde. The brains were removed and dissected on ice, then post-fixed in the same fixative at 4°C overnight. The hippocampus was hand-picked and cut into blocks of ∼1 mm^3^. Then, the blocks were placed into the 1% osmium tetroxide for 2 h at 4°C. After post-fixation, the blocks were rinsed in 0.01 mol/L phosphate buffer solution (PBS) (pH 7.4) three times and dehydrated in a graded series of ethanol and then in acetone. Thin sections were embedded in Poly/Bed 812 embedding kit (Polysciences, Warrington, PA, United States), cut on an Ultracut E ultramicrotome (Reichert, Buffalo, NY, United States), and stained with uranyl acetate and lead citrate. The sections were examined in a Hitachi H7650 transmission electron microscope (Hitachi High-Tech, Fukuoka, Japan), operating at an accelerating voltage of 80.0 kV. Images were captured with the AMT Camera System.

### Cell Culture and Reagents

Human neuroblastoma SH-SY5Y cells were cultured in DMEM/F12 medium (Gibco, Thermo Fisher Scientific, United States) at 37°C with 5% CO_2_. The medium was supplemented with 10% fetal bovine serum (Gibco, Thermo Fisher Scientific, United States), 100 U/mL penicillin, 100 μg/mL streptomycin (Gibco, Thermo Fisher Scientific, United States).

### Preparation of Aged Aβ_1__–__42_

Aβ_1__–__42_ peptide (Chinese Peptide Co., Hangzhou, China) was dissolved in distilled water at a concentration of 5 mM and incubated at 4°C for 7 days for aggregation. The aged Aβ_1__–__42_ was stored frozen at −20°C until use.

### Aβ_1–42_ and Drug Treatment

SH-SY5Y cells were incubated for 24 h at 37°C, then the medium was replaced with serum-free DMEM/F12 medium supplement with Aged Aβ_1__–__42_ at 50 μM for 8 h. ECH is dissolved in PBS, filtered and sterilized by 0.2 μm filter. After Aβ_1__–__42_ treatment, ECH was added to the medium to a final concentration of 10 mM, and the cells were incubated at 37°C for 24 h for subsequent assay.

### BACE1 Degradation Assay

For hippocampus primary neuron culture, day 0–1 pups of APP/PS1 mice were collected and the tails were clipped for genotype identification with PCR. Hippocampi of APP/PS1 pups were washed and dissected in cold HBBS (without Ca2+ and Mg2+), dissociated with 0.05% trypsin at 37°C for 15 min, and then triturated with a Pasteur pipette gently in Neurobasal A medium (Gibco, Thermo Fisher Scientific, United States). After non-dispersed tissue settled for 5 min, the supernatant was centrifuged for 5 min at 200 × *g*. The pellet was gently resuspended in neuron culture medium (Neurobasal A medium containing 2% B27 and 0.5 mM L-glutamine). Then neuron suspension was plated onto poly-D-lysine (Sigma-Aldrich)-coated 6-well-plate. After 24 h, the culture medium was replaced by a fresh medium with or without ECH/MG132 (Sigma-Aldrich, United States), the inhibitor of proteasomes, and incubated for 12 h. Then the neurons were collected and extracted cellular protein for Western blotting assay.

### Immunofluorescence and Image Analysis

SH-SY5Y cells were treated to different experimental conditions on coverslips of 30000 cells in 24-well plates, fixed with 4% paraformaldehyde and 4% sucrose in PBS for 20 min. Then the cells were permeabilized with 0.2% Triton X-100 (v/v in PBS) for 5 min and blocked with 0.2% gelatin. Then the coverslips were incubated with selected primary and secondary antibodies in blocking buffer. Primary antibodies are polyclonal anti-FLNA (1:100, #4762S, Cell Signaling Technology, Danvers, MA, United States) and monoclonal anti-PERK (1:50, sc-377400, Santa Cruz, Dallas, TX, United States); Secondary antibodies included goat anti-rabbit IgG- rhodamine (1:200, ZF-0316, ZSGB-BIO Co., Beijing, China) and goat anti-mouse IgG-Alexa Fluor^®^ 488 (1:200, ZF-0512, ZSGB-BIO Co., Beijing, China).

Serial 4-μm coronal paraffin-embedded sections of the hippocampus of mice were used for immunofluorescence assay. After deparaffinization, rehydration and antigen retrieval, paraffin sections were incubated with mouse monoclonal anti-F-actin (1:100, ab130935, Abcam, United Kingdom) and rabbit polyclonal G-actin antibodies (1:100, ab194952, Abcam, United Kingdom) overnight. Secondary antibodies included goat anti-mouse IgG- rhodamine (1:200, ZF-0313, ZSGB-BIO Co., Beijing, China) and goat anti-rabbit IgG-Alexa Fluor^®^ 488 (1:200, ZF-0511, ZSGB-BIO Co., Beijing, China). Digital images of SH-SY5Y cells on coverslips and mouse hippocampus sections were captured with Olympus IX71 microscope and analyzed with Image J 1.46r (NIH, United States).

### F-actin/G-actin Fraction Assay

F-actin/G-actin ratio in mice was determined with G-actin/F-actin In Vivo Assay Kit (#BK037, Cytoskeleton, Inc, United States) according to the manufacturer’s instructions. Briefly, mouse brain tissue was homogenized in the lysis buffer and incubated at 37°C for 10 min. The lysates were centrifuged at 350 × *g* for 5 min, and the supernatants were centrifuged at 100,000 × *g* at 37°C for 1 h. The precipitate is F-actin, and the supernatant is G-actin. Transfer the supernatant containing G-actin to a new tube. Add depolymerization buffer to the precipitate containing F-actin and incubate on ice for 1 h. The extracts containing G-actin and F-actin were assayed by Western blot respectively.

### MicroScale Thermophoresis (MST) Assay

The mouse and human PERK proteins (Sino Biological Inc., Beijing, China) were labeled with the Monolith NT Protein Labeling Kit RED (NanoTemper Technologies, Munich, Germany) according manufacture’s protocol. Labeled-mouse and -human PERK were used at a concentration of 25 nM in PBS (pH 7.2) containing 0.05 (v/v) % Tween-20. ECH at 1000 μM in PBS was serially diluted in a 1:1 ratio into 16 gradient concentrations. The binding reaction systems of ECH with labeled-mouse or -human PERK were incubated for 10 min at room temperature avoiding light, then loaded into hydrophilic capillaries (NanoTemper). The measurements were performed on a NanoTemper Monolith NT.115 instrument (NanoTemper) at 40 % MST power, 30-s Laser-On time, and 5-s Laser-Off time.

### Molecular Docking Analysis

The X-ray structures of human PERK (EIF2AK3, PDB ID: 4X7J) and mouse PERK (EIF2AK3, PDB ID: 3DQ2) were obtained from the RCSB protein database. The structures of ECH were optimized by HyperChem 7.0 software (Hypercube, Gainesville, FL, United States). A molecular docking study was carried out using Glide of Schrödinger molecular modeling suite (version 2015-2) (Schrödinger, Cambridge, MA, United States) running on a Linux 64-bit operating system. Glide XP visualizer was used for the analysis of protein-ligand interactions, including glide score, glide energy, hydrogen bond interactions, and other interactions. The three-dimensional and two-dimensional analyses were done in Discovery Studio 2.5 (Biovia, San Diego, CA, United States).

### Statistical Analyses

Statistical analyses were performed using the SPSS program (version 20.0 for windows) (IBM, Armonk, NY, United States). Values were expressed as the means ± SEM. Eighteen mice were used for each behavioral assay, 5–6 mice were used for each histological study, and 6 mice were used for each biochemical study. The escape latencies in the Morris water maze test were analyzed using two-way repeated-measures ANOVA followed by Fisher’s protected least significant difference (LSD) test for *post hoc* comparisons. The Aβ-plaque load was evaluated via analysis of variance (ANOVA) to compare four groups followed Tukey’s test for *post hoc* multiple comparison between-group analyses. The remaining biochemical data were analyzed using one-way ANOVA followed by the LSD test for *post hoc* comparisons. The level of significance was set at *P* ≤ 0.05.

## Results

### ECH Improves Cognitive Function in 2 × Tg-AD Mice

**FIGURE 2:**
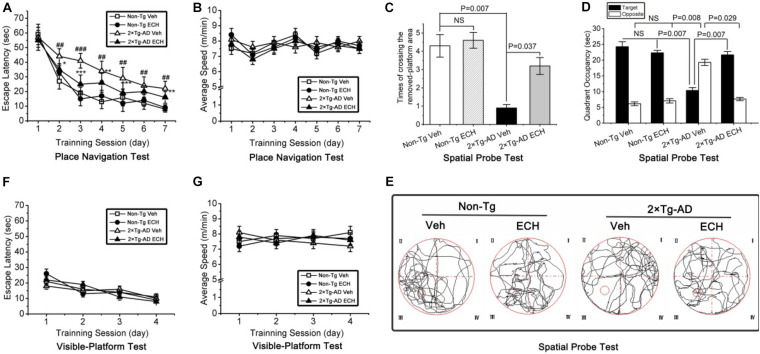
ECH treatment improves spatial learning and memory in 10-month-old 2 × Tg-AD mice in the Morris water maze test. **(A)** In the place navigation test, the vehicle-treated 2 × Tg-AD mice showed significantly longer escape latencies compared with vehicle-treated Non-Tg mice (^##^*P* < 0.01, ^###^*P* < 0.001, vehicle-treated 2 × Tg-AD mice vs. vehicle-treated Non-Tg mice), and ECH treatment reduced escape latency in 2 × Tg-AD mice remarkably (*P < 0.05, **P < 0.01, and ***P < 0.001, ECH-treated 2 × Tg-AD mice vs. vehicle-treated 2 × Tg-AD mice). **(B)** No significant differences were detected in the average swimming speed among the 4 groups during the place navigation test (*P* = 0.516). During the spatial probe test, **(C)** the number of crossings of the area of the removed platform and **(D)** time spent in the target vs. opposite quadrant were recorded; vehicle-treated 2 × Tg-AD mice showed significantly inferior performance than ECH-treated 2 × Tg-AD mice and vehicle- and ECH-treated Non-Tg mice. **(E)** Representative images of the route of travel during the spatial probe test were recorded. No significant differences were detected in **(F)** the escape latency (*P* = 0.379) or **(G)** the average swimming speed (*P* = 0.725) among the 4 experimental groups during the visible-platform test. All data are presented as the mean ± SEM (*n* = 18 mice per group). Non-Tg, non-transgenic littermates; 2 × Tg-AD, APPswe/PS1dE9 mice; Veh, vehicle (normal saline); ECH, echinacoside; NS, no significant difference.

Spatial learning and memory deficits emerge and progressively worsen with age in 2 × Tg-AD mice (Trinchese et al., 2004; Ruan et al., 2009; Zhu et al., 2013b), and the Morris water maze test is usually performed to evaluate this process (Zhu et al., 2013b; Du et al., 2016). To evaluate the protective effects of ECH on cognitive impairment in 2 × Tg-AD mice, we measured the spatial learning and memory of 2 × Tg-AD mice with the Morris water maze test. After 6 months of intervention, the spatial learning ability was assessed by the escape latency across the 7-day acquisition training period in the place navigation test. As shown in Figure 2A, the mean escape latency declined progressive during the 7-day place navigation test, and there was a significant difference among the four groups: day, *F*(6,476) = 23.424, *P* = 0.001; treatment, *F*(3,476) = 8.853, *P* = 0.007; day by treatment interaction, *F*(18,476) = 4.749, *P* = 0.396. Moreover, vehicle-treated 2 × Tg-AD mice showed longer escape latencies during the acquisition training period (from day 2 to day 7) relative to the vehicle-treated Non-Tg mice (day 1, *P* = 0.146; day 2, *P* = 0.007; day 3, *P* = 0.001; day 4, *P* = 0.004; day 5, *P* = 0.008; day 6, *P* = 0.006; day 7, *P* = 0.008). This result suggested that 2 × Tg-AD exhibited significant cognitive impairment. In contrast, except for day 1 and day 6, ECH-treated 2 × Tg-AD mice showed a significant decrease in the escape latency compared with the vehicle-treated 2 × Tg-AD mice (day 1, *P* = 0.391; day 2, *P* = 0.008; day 3, *P* = 0.001; day 4, *P* = 0.006; day 5, *P* = 0.009; day 6, *P* = 0.068; day 7, *P* = 0.006). Furthermore, the concurrent recorded average swimming speed reflected no significant differences among the 4 groups during the place navigation test: *F*(3,476) = 11.071, *P* = 0.516. These results implied that ECH treatment did not significantly induce any physical fitness changes in 2 × Tg-AD or Non-Tg mice (Figure 2B).

Then, the spatial probe test was performed 24 h after the final day of the place navigation test (Day 8). As shown in Figures 2C,E, one-way ANOVA revealed a significant difference in the number of crossings of the area of the removed platform among the four groups of mice [*F*(3,68) = 8.882, *P* = 0.006]. Moreover, vehicle-treated 2 × Tg-AD mice crossed above the area of the removed platform less frequently compared to the vehicle-treated Non-Tg mice (*P* = 0.007). However, ECH-treated 2 × Tg-AD mice crossed the area of the removed platform more often than the vehicle-treated 2 × Tg-AD mice (*P* = 0.037).

As shown in Figure 2D, the time spent in the target and opposite quadrants showed significant differences among the four groups of mice: the target quadrant [*F*(3,68) = 14.835, *P* = 0.004]; opposite [*F*(3,68) = 31.729, *P* = 0.001]. Moreover, compared to the vehicle-treated Non-Tg mice, vehicle-treated 2 × Tg-AD mice spent less time searching for the platform in the target quadrant (*P* = 0.007) and more time in the opposite quadrant (*P* = 0.008). In contrast, ECH-treated 2 × Tg-AD mice spent more time in the target quadrant (*P* = 0.007) and less time in the opposite quadrant (*P* = 0.029) compared with the vehicle-treated 2 × Tg-AD mice. As shown in Figure 2E, the results are supported by representative images of the movement trajectory during the spatial probe test, suggesting that ECH-treated 2 × Tg-AD mice appeared more frequently in and around the platform area compared to vehicle-treated 2 × Tg-AD mice.

In addition, there were no significant differences in the escape latency [*F*(3,272) = 37.820, *P* = 0.379] (Figure 2F) or the average swimming speed [*F*(3,272) = 62.06, *P* = 0.725] (Figure 2G) among the 4 experimental groups during the visible-platform test. This result indicated that the spatial learning and memory deficits observed in the 2 × Tg-AD mice were not attributable to non-cognitive factors (i.e., motor, visual, and motivation abnormalities), and the effect of ECH on improving memory in 2 × Tg-AD mice did not affect the factors mentioned above. Interestingly, there was no significant difference in the escape latency (Figure 2A), the frequency of crossing the area of the removed platform (Figure 2C; *P* = 0.369) or time spent in the target (*P* = 0.761) or opposite quadrant (Figure 2D; *P* = 0.604) between the vehicle- and ECH-treated Non-Tg mice, which demonstrated that ECH itself did not directly have a significant effect on cognition in Non-Tg mice. Simultaneously, in accordance with previous research (Zhao et al., 2010; Zhang et al., 2017), the daily dose of ECH (30 mg/kg b.w) for each mouse was safe and effective. Taken together, ECH intervention strongly ameliorated the spatial learning and memory impairments in 2 × Tg-AD mice.

### ECH Reduces Cerebral Aβ-Positive Plaque Load and Aβ Production in 2 × Tg-AD Mice

**FIGURE 3:**
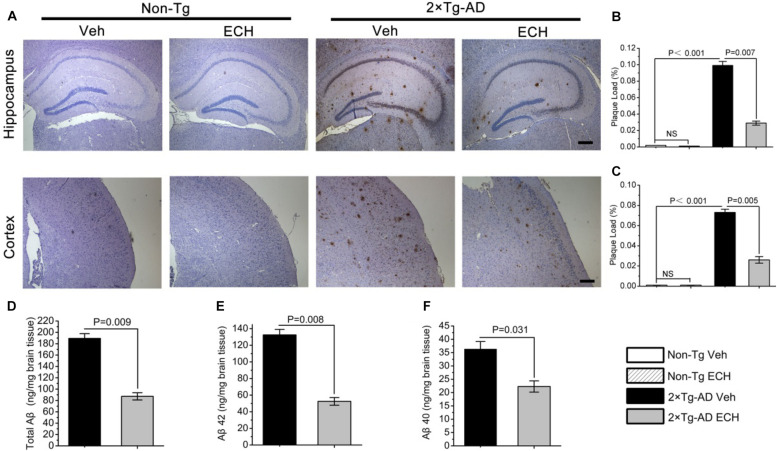
ECH reduces Aβ-positive plaque load and Aβ production in the hippocampus and cortex of 2 × Tg-AD mice. Mouse brain sections were stained with anti-Aβ antibody 6E10. **(A)** Images of Aβ immunoreactivity in hippocampus and cortex of the indicated groups of mice were photographed with an IX71 microscope. Scale bar = 200 μm. Aβ-positive plaque load in the **(B)** hippocampus and **(C)** cortex of mice were analyzed with Image-Pro Plus 6.0 software, and the plaque load was defined as the percentage of the sum of Aβ deposit areas compared with the total section area (Non-Tg Veh, *n* = 5; Non-Tg ECH, *n* = 6; 2 × Tg-AD Veh, *n* = 5; 2 × Tg-AD ECH, *n* = 5). The levels of **(D)** total Aβ, **(E)** Aβ_1__–__42_, and **(F)** Aβ_1__–__40_ in brain homogenates of 2 × Tg-AD mice administered vehicle or ECH were measured by enzyme-linked immunosorbent assay (ELISA), and the values are presented as nanogram per milligram of brain tissue. All data are presented as the mean ± SEM (*n* = 6 mice per group). Non-Tg, non-transgenic littermates; 2 × Tg-AD, APPswe/PS1dE9 mice; Veh, vehicle (normal saline); ECH, echinacoside; NS, no significant difference.

Given that extracellular Aβ deposition, the neuropathological hallmark of AD, plays a key role in cognitive deficits in AD (Rattanajarasroj and Unchern, 2010), and ECH can improve the cognition of 2 × Tg-AD mice, we assessed whether ECH could reduce Aβ-plaque load in the hippocampus and cortex in 2 × Tg-AD mice. Figure 3A presents images of Aβ-plaque immunoreactivity in the hippocampus and cortex of the 4 groups, showing that 2 × Tg-AD mice at 10 months of age developed obvious Aβ-plaque deposition in both hippocampus and cortex. This AD-like pathogenesis was consistent with previous reports (Capell et al., 2000; Luo et al., 2016). Compared with vehicle-treated Non-Tg mice, quantitative analysis revealed that the vehicle-treated 2 × Tg-AD mice exhibited a significantly greater Aβ-plaque load in the hippocampus (Figure 3B, *P* < 0.001) and cortex (Figure 3C, *P* < 0.001). ECH treatment markedly decreased the hippocampal (Figure 3B, *P* = 0.007) and cortical Aβ-plaque load (Figure 3C, *P* = 0.005) of 2 × Tg-AD mice compared to those of vehicle-treated 2 × Tg-AD mice, indicating that ECH could significantly reduce the extracellular accumulation of Aβ in both hippocampus and cortex of 2 × Tg-AD mice.

The content of soluble Aβ, including Aβ42 and Aβ40, reflects the production of Aβ and is closely related to the formation of Aβ-plaques. To examine whether the decrease in Aβ-plaque with ECH treatment is attributable to the effects of ECH on Aβ production, we assessed the level of cerebral total Aβ, Aβ42, and Aβ40 in the vehicle- and ECH- treated 2 × Tg-AD mice by ELISA. In accord with the Aβ-plaque load shown in Figures 2A–C, the levels of total Aβ (Figure 3D, *P* = 0.009), Aβ42 (Figure 3E, *P* = 0.008), and Aβ40 (Figure 3F, *P* = 0.031) in brain homogenates were significantly reduced in the ECH-treated 2 × Tg-AD mice compared with the vehicle-treated mice, indicating that ECH reduced cerebral Aβ production in 2 × Tg-AD mice.

### ECH Represses the Translation and Enzymatic Activity of BACE1 in 2 × Tg-AD Mice

**FIGURE 4:**
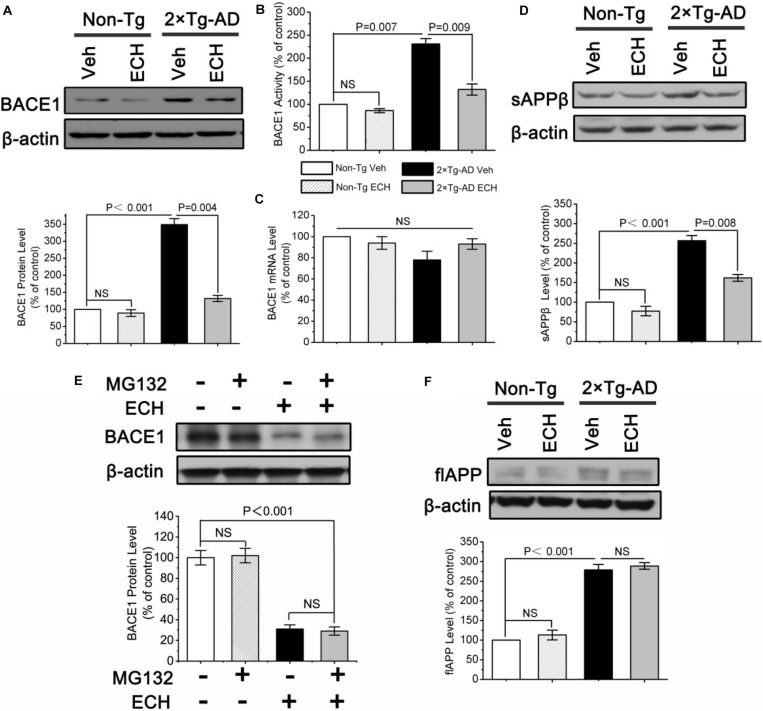
ECH downregulates the protein level and enzymatic activity of BACE1 in 2 × Tg-AD mice. **(A)** The protein level of BACE1 in brain homogenates of mice was assayed by Western blot. **(B)** BACE1 activity was fluorimetrically monitored as described under section “Materials and Methods” in the brain homogenates of the indicated groups of mice. **(C)** The effects of ECH on *BACE1* gene transcription were evaluated by real-time RT-PCR. Effects of ECH on **(D)** Protein levels of the soluble APP β fragments (sAPPβ), **(E)** ubiquitination degradation of BACE1 and **(F)** full-length APP (flAPP) level were determined by Western blot. All data are presented as the mean ± SEM (*n* = 6 mice per group). Non-Tg, non-transgenic littermates; 2 × Tg-AD, APPswe/PS1dE9 mice; Veh, vehicle (normal saline); ECH, echinacoside; NS, no significant difference.

BACE1 is a key rate-limiting enzyme for APP processing to produce Aβ that catalyzes the initial cleavage of the APP and gives rise to Aβ (Vassar and Kandalepas, 2011; Yan and Vassar, 2014). To explore whether ECH prevents Aβ generation by downregulating BACE1, we first tested the effects of ECH on the transcription, translation and enzymatic activity of BACE1. As shown in Figure 4A, the protein level of BACE1 was significantly increased in the brain of vehicle-treated 2 × Tg-AD mice relative to the control Non-Tg mice (*P <* 0.001), consistent with other studies (O’connor et al., 2008; Kim et al., 2018). ECH treatment significantly decreased BACE1 protein expression in 2 × Tg-AD mice (Figure 4A, *P* = 0.004). Consistent with this result, the BACE1 activity among the 4 groups revealed the same trend as the BACE1 protein level (Figure 4B). Notably, there was no significant difference in the BACE1 protein level or activity between vehicle- and ECH-treated Non-Tg mice (Figures 4A,B), suggesting that ECH had no significant effect on the protein level or enzyme activity of BACE1 in Non-Tg mice.

ECH decreased BACE1 protein expression, possibly due to inhibition of the transcription of the *BACE1* gene. Therefore, we determined the effect of ECH on *BACE1* transcription by measuring the *BACE1* mRNA level with real-time RT-PCR. Consistent with previous reports (Preece et al., 2003; O’connor et al., 2008; Kim et al., 2018), the results (Figure 4C) showed that there was no significant difference in *BACE1* mRNA among the four indicated groups, even though the mRNA level in ECH-treated 2 × Tg-AD mice exhibited an increasing trend compared with vehicle-treated 2 × Tg-AD. These results conclusively demonstrate that ECH-induced BACE1 reduction in 2 × Tg-AD mice was due to the posttranscriptional rather than the transcriptional downregulation of *BACE1*.

The cleavage of APP by BACE1 produces a soluble APP fragment β (sAPPβ). To examine whether the reduced amyloidosis in the presence of ECH was caused by the reduction in APP metabolism through BACE1, we next examined the effect of ECH on the secretion of sAPPβ by Western blot. As shown in Figure 4D, ECH treatment significantly reduced sAPPβ in the brains of 2 × Tg-AD mice relative to the vehicle-treated 2 × Tg-AD mice (*P* = 0.008), which was consistent with the results of BACE1 protein level and activity, strongly suggesting that ECH decreases Aβ by modulating APP processing through the inhibition of the BACE1-mediated cleavage of APP for the amyloidogenic pathway.

Promoting ubiquitination degradation may be another reason for the decrease in BACE1 protein level. To further study the effect of ECH on the ubiquitination degradation of BACE1, we utilized the proteasome inhibitor MG132 to process hippocampal primary neurons of 2 × Tg-AD mice. As shown in Figure 4E, MG132 did not affect BACE1 levels, regardless of ECH intervention. It indicated that ECH reduced the protein level of BACE1 not by promoting its ubiquitination degradation.

A potential concern about our data is that ECH-decreased Aβ production may be due to the reduction in the APP level. In view of the fact that APP protein has many normal physiological functions (Dawkins and Small, 2014; Montagna et al., 2019), depressing its production will result in some adverse consequences. We sought to definitively exclude this possibility by assessing the full-length APP (flAPP) protein level in the brain of mice with or without ECH treatment. As shown in Figure 4F, there was markedly increased expression of flAPP in the brain of the 2 × Tg-AD mice relative to the control Non-Tg mice due to the overexpression of *APP* gene under the constitutively active CMV promoter (*P* < 0.001). However, as shown in Figure 4F, there are no significant differences in the flAPP level between ECH- and vehicle-treated Non-Tg mice (*P* = 0.486) or 2 × Tg-AD mice (*P* = 0.635), suggesting that ECH intervention itself had little effect on APP synthesis in the brain of 2 × Tg-AD mice. Therefore, ECH does not inhibit Aβ generation by reducing APP production.

### ECH Has Little Effect on Expression or Enzymatic Activity of α- or γ-Secretase in 2 × Tg-AD Mice

**FIGURE 5:**
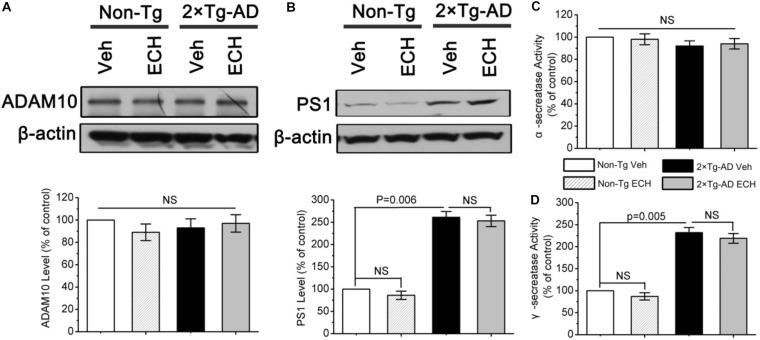
Effects of ECH on α- and γ-secretase in 2 × Tg-AD mice. The levels of **(A)** A-disintegrin and metalloproteinase 10 (ADAM10) and **(B)** presenilin 1 (PS1) from the brain of mice were determined by Western blot. The enzymatic activity of **(C)**α-secretase and **(D)**γ-secretase in the brains of the indicated groups of mice were fluorimetrically monitored as described under section “Materials and Methods.” All data are expressed as the percentage of vehicle-treated Non-Tg mice and are presented as the mean ± SEM (*n* = 6–8 mice per group). Non-Tg, non-transgenic littermates; 2 × Tg-AD, APPswe/PS1dE9 mice; Veh, vehicle (normal saline); ECH, echinacoside; NS, no significant difference.

ECH-induced Aβ reduction may also result from the upregulation of α-secretase or inhibition of γ-secretase. ADAM10 and PS1 are the catalytic subunits of α- and γ-secretase, respectively (Fahrenholz et al., 2000; Meckler and Checler, 2016). To determine whether ECH inhibits Aβ production by regulating the level and activity of α- and γ-secretase, we assessed the levels of catalytic subunits and enzymatic activities of α- and γ-secretase in mice with or without ECH treatment. As shown in Figures 5A,C, ECH treatment did not show a significant effect on the level of ADAM10 or the enzymatic activity of α-secretase in 2 × Tg-AD mice or Non-Tg mice. These results demonstrated that ECH did not influence the level or the activity of α-secretase in either 2 × Tg-AD mice or Non-Tg mice. PS1 was significantly elevated in 2 × Tg-AD mice compared to Non-Tg mice (*P* = 0.006), which was consistent with the overexpression of the PS1 mutant gene in 2 × Tg-AD mice (Kurt et al., 2001). However, there was no significant difference in the level of PS1 between ECH- and vehicle-treated 2 × Tg-AD mice (Figure 5B, *P* = 0.379). In accord with this result, γ-secretase activity among the 4 groups revealed the same trend as the PS1 protein level (Figure 5D). Therefore, the inhibitory effect of ECH on Aβ production in 2 × Tg-AD mice was not due to the inhibition of the γ-secretase.

In summary, the reduction in Aβ production induced by ECH treatment was most likely attributed to its inhibitory effect on the amyloidogenic cleavage of APP by reducing the BACE1 protein level exclusively through a posttranscriptional mechanism, without any effect on the regulation of BACE1 transcription, APP synthesis, ADAM10, PS1 expression, or the enzymatic activity of α- or γ-secretase.

### ECH Depresses ERS via the PERK/eIF2α-Mediated Pathway in 2 × Tg-AD Mice

**FIGURE 6:**
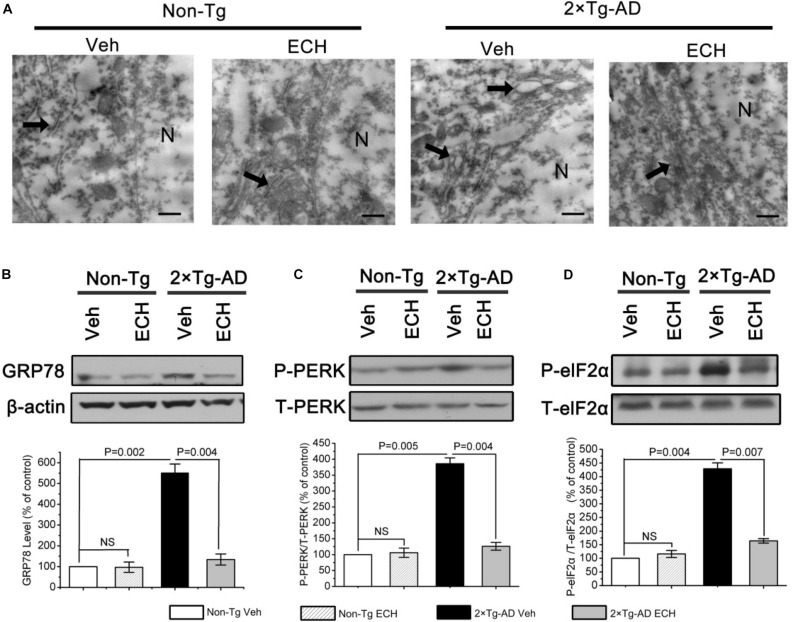
ECH depresses ERS via PERK/eIF2α-mediated pathway in 2 × Tg-AD mice. **(A)** Hippocampal tissues were observed under the transmission electron microscope (TEM), and the morphological changes in neuronal ER are indicated with thick arrows. Scale bar = 500 nm. The levels of GRP78 **(B)**, phosphorylated, and total eIF2α **(C)** and PERK **(D)** from brain homogenates of the indicated groups of mice were detected by Western blot. The relative levels of phosphorylated proteins were normalized to the total protein content and are expressed as the percentage of Non-Tg Veh mice. All data are presented as the mean ± SEM (*n* = 6–8 mice per group). Non-Tg, non-transgenic littermates; 2 × Tg-AD, APPswe/PS1dE9 mice; Veh, vehicle (normal saline); ECH, echinacoside; NS, no significant difference; N, nucleus.

ERS is induced by excessive Aβ accumulation and results in the elevated translation of BACE1 via PERK/eIF2α activation in AD transgenic mice and patients with AD (O’connor et al., 2008; Hetz and Mollereau, 2014; Bell et al., 2016). Based on our finding that ECH decreased Aβ production by depressing BACE1 translation, we speculated that ECH treatment would inhibit Aβ-induced ERS and posttranscriptionally reduce the expression of BACE1 through inhibiting the activation of the PERK/eIF2α pathway in 2 × Tg-AD mice. To determine whether ECH could suppress ERS induced by overproduced Aβ in 2 × Tg-AD mice, we first performed transmission electron microscopy (TEM) analysis to observe the ultrastructure of ER in hippocampal neurons of mice with or without ECH treatment. The rough ER maintained its normal structure in Non-Tg mice (Figure 6A, left two panels), while it exhibited significant swelling and degranulation in vehicle-treated 2 × Tg-AD mice (thick arrows indicate ER structure in Figure 6A). Accordingly, the abnormal accumulation of unfolded proteins in the ER was very easy to identify. As shown in Figure 6A, ECH significantly ameliorated the morphological abnormalities of ER in the hippocampus of 2 × Tg-AD mice. To further confirm whether ERS was activated in the brain of 2 × Tg-AD mice and the effect of ECH on it, we next assessed the level of glucose-regulated protein 78 (GRP78) by Western blot because GRP78 is the earliest marker of ERS and appears to be the most sensitive in 2 × Tg-AD mice (Ron and Walter, 2007). As shown in Figure 6B, GRP78 increased dramatically in vehicle-treated 2 × Tg-AD mice compared to Non-Tg control (*P* = 0.002). Compared with vehicle-treated 2 × Tg-AD mice, ECH markedly reduced the levels of GRP78 in 2 × Tg-AD mice (*P* = 0.004). Taken together, we conclude that there was an obvious increase in ERS in the brains of 2 × Tg-AD mice and that ECH treatment effectively depressed this increased ERS.

Given that the increase in GRP78 results in the activation of PERK/eIF2α in the AD brain during ERS and that it induces the UPR (Chang et al., 2002; O’connor et al., 2008; Ohno, 2014), we further explored the molecular mechanism by which ECH ameliorates ERS using Western blot to evaluate the phosphorylation of PERK and eIF2α in 2 × Tg-AD mice. As expected, the phosphorylation of PERK (*P* = 0.005) and eIF2α (*P* = 0.004) was increased markedly in vehicle-treated 2 × Tg-AD mice. In contrast, ECH significantly decreased the phosphorylation of PERK (*P* = 0.004) and eIF2α (*P* = 0.007) in 2 × Tg-AD mice (Figures 6C,D). These findings indicated that ECH repressed ERS through effectively inhibiting PERK/eIF2α activation in 2 × Tg-AD mice.

### ECH Promotes PERK and FLNA Combination and ER-PM Contacts

**FIGURE 7:**
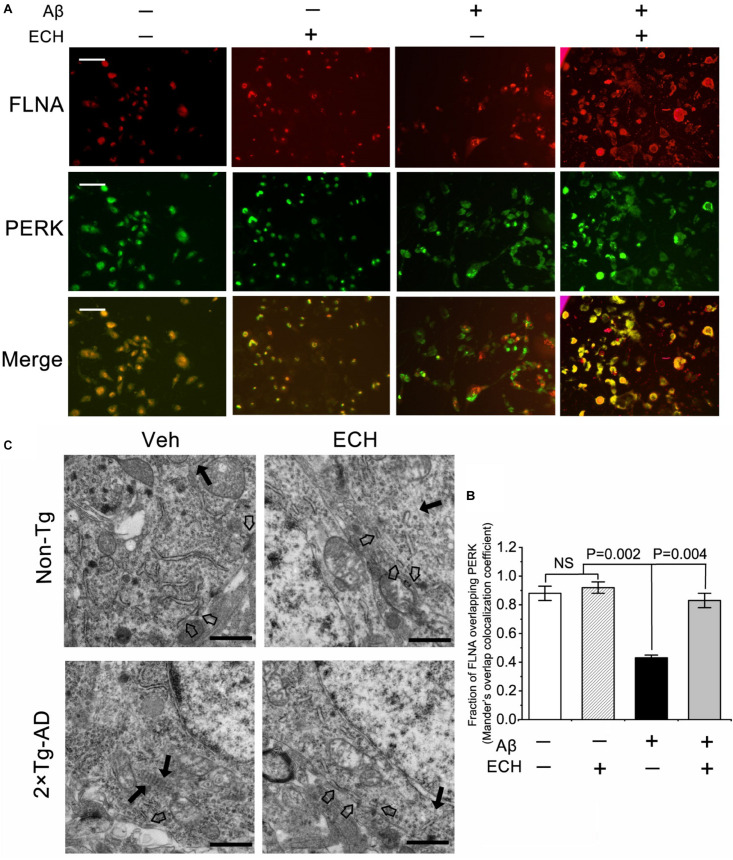
ECH promotes the combination of PERK and FLNA and ER-PM contacts. **(A)** Aβ_1__–__42_-treated SH-SY5Y cells were incubated with or without ECH. Immunofluorescence assay showed the colocalization of FLNA (red) and PERK (green), scale bar = 50 μm. **(B)** Mander’s overlap colocalization analysis of data represented in **(A)** (mean ± SEM; *n* = 3, 30 cells were analyzed per condition). **(C)** Representative electron micrographs of hippocampal tissues of Non-Tg mice and 2 × Tg-AD mice treated with or without ECH. Black arrows denote F-actin, and hollow arrow denoted ER-PM contact sites, scale bar = 1.0 μm. FLNA, filamin-A; Non-Tg, non-transgenic littermates; 2 × Tg-AD, APPswe/PS1dE9 mice; Veh, vehicle (normal saline); ECH, echinacoside; NS, no significant difference.

Phosphorylation of PERK could depress its interaction with FLNA, thus results in F-actin accumulation at cell edges and perturbs the formation of ER-PM contacts. Based on the above findings that ECH inhibits PERK phosphorylation, we further investigate whether ECH can promote the interaction between PERK and FLNA and in turn regulate F-actin remodeling. The immunofluorescent assay revealed that the combination of PERK with FLNA decreased due to PERK phosphorylation induced by Aβ in Aβ_1–42_-treated SH-SY5Y cells as compared to control (Figures 7A,B). After 24 h incubation with ECH, PERK- FLNA interaction was reinforced. TEM of mouse hippocampus showed that in Non-Tg mice (treated with Veh or ECH) F-actin evenly distributed in neuron. In contrast, 2 × Tg-AD mice treated with vehicle, along with the more peripheral accumulation of F-actin, displayed reduced ER-PM contacts. 2 × Tg-AD mice treated with ECH exhibited more scattered distribution of F-actin fiber and more ER-PM contact sites (Figure 7C).

### ECH Modulates F-actin Remodeling and the Ratio of F-actin/G-actin

**FIGURE 8:**
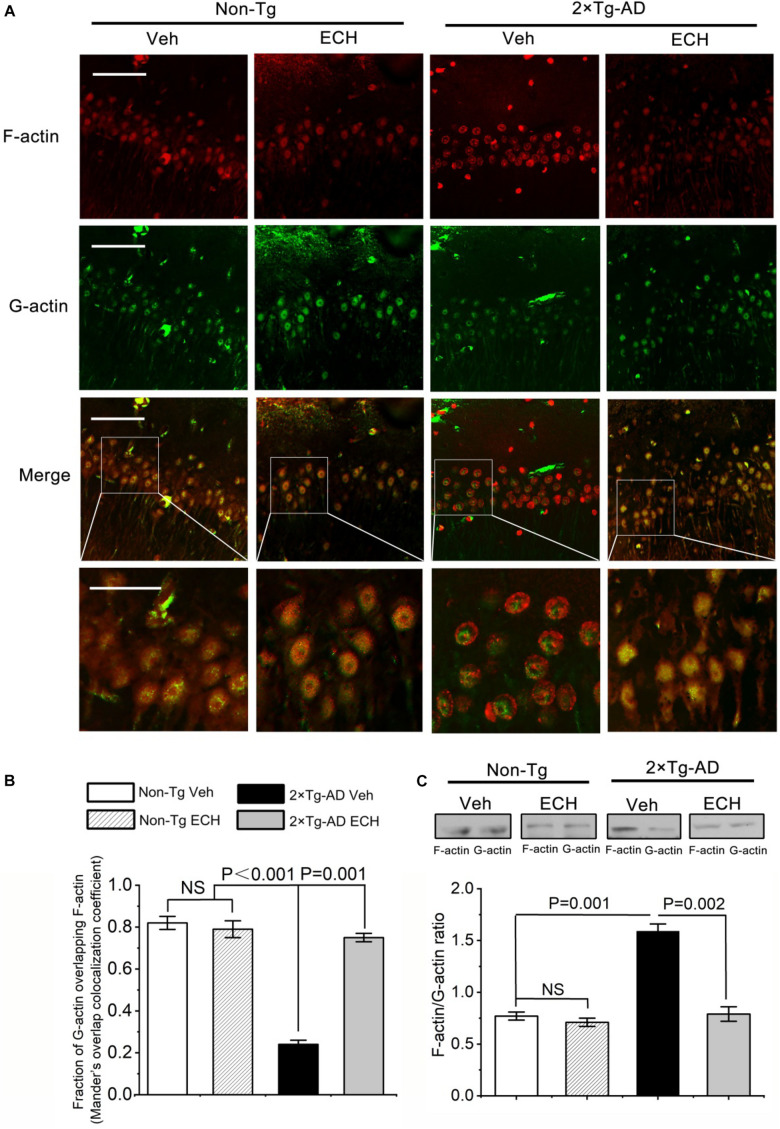
ECH modulates the ratio of F-actin and G-actin. **(A)** representative images of F-actin and G-actin in the hippocampus of Non-Tg mice and 2 × Tg-AD mice administrated with or without ECH. Immunofluorescence assay showed the colocalization of F-actin (red) and G-actin (green), scale bar = 50 μm (Upper three-row panel) and scale bar = 20 μm (Fourth-row panel). **(B)** Mander’s overlap colocalization analysis of data represented in **(A)**. (mean ± SEM; n = 3, 35 cells were analyzed per condition). **(C)** Cerebral F-actin/G-actin ratio in Non-Tg mice and 2 × Tg-AD mice administrated with or without ECH. Non-Tg, non-transgenic littermates; 2 × Tg-AD, APPswe/PS1dE9 mice; Veh, vehicle (normal saline); ECH, echinacoside; NS, no significant difference.

In hippocampus neurons of Non-Tg mice treated with or without ECH, F-actin exhibited enrichment distribution at the cell edges. However, F-actin was more peripheral in 2 × Tg-AD mice. Fluorescent images of homogeneous G-actin in Non-Tg mice treated with or without ECH showed a partially overlapping with F-actin. On the contrary, in 2 × Tg-AD mice administrated with Vehicle, the distribution area of G-actin is very different from that of F-actin which is more distributed on the edge of the cell, while G-actin is more distributed in the center area of the cell. ECH can significantly regulate the remodeling and redistribution of F-actin in 2 × Tg-AD mice (Figures 8A,B). Since the abnormal distribution of F-actin is closely related to its polymerization which was reflected by the ratio of F-actin/G-actin, we then investigated the effect of ECH on the F-actin/G-actin ratio. As shown in Figure 8C, 2 × Tg-AD mice administrated with Vehicle showed disturbance ratio of F-actin/G-actin, and ECH could modulate and ameliorate F-actin polymerization status.

### Analysis of Binding Models of ECH and PERK

**FIGURE 9:**
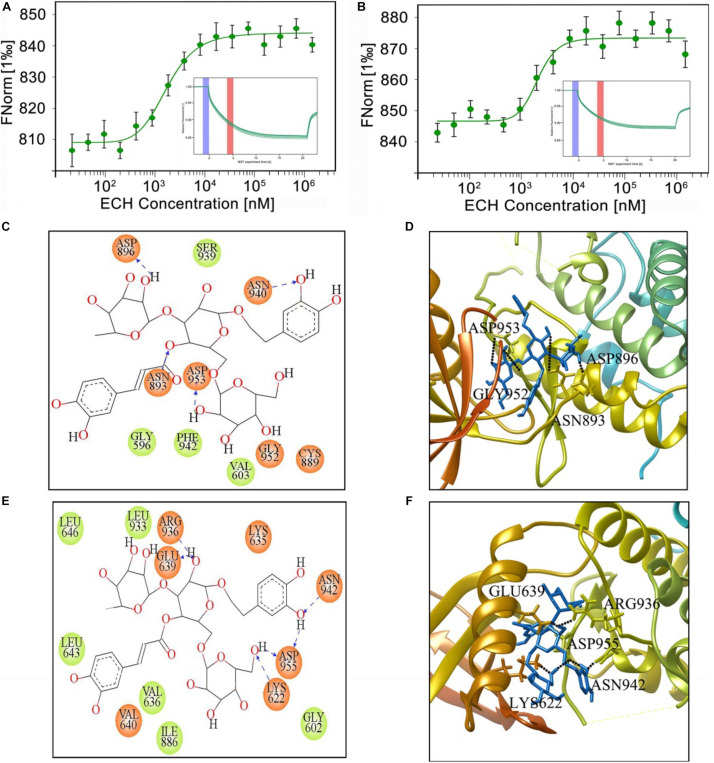
MST measurement and binding mode of ECH and mPERK or hPERK. MST binding cure (*n* = 3) and MST curve (inset) of ECH binding to mPERK **(A)** or hPERK **(B)**. **(C)** Two-dimensional and **(D)** three-dimensional binding model between mouse PERK (EIF2AK3, PDB ID: 3DQ2) and ECH. **(E)** Two-dimensional and **(F)** three-dimensional binding models between human PERK (EIF2AK3, PDB ID: 4X7J) and ECH. The black dotted lines represent the hydrogen bond. The blue sticks in the three-dimensional binding model **(D,F)** represent ECH.

PERK is an ER transmembrane protein with a cytosolic kinase domain. During ERS, PERK is activated through dimerization and autophosphorylation (Devi and Ohno, 2014). The findings in the present study indicated that ECH could inhibit PERK phosphorylation in 2 × Tg-AD mice, so we hypothesized that ECH binds to the PERK molecule to prevent its phosphorylation. To test this hypothesis, we performed MST assay to evaluate the affinity of ECH to mouse PERK (mPERK) or human (hPERK). As shown in Figures 9A,B, ECH exhibited a high affinity to mPERK and hPERK. The K_*d*_ of ECH binding to mPERK or hPERK were 0.851 ± 0.081 μM and 0.635 ± 0.074 μM, respectively.

To further analyze the way by which ECH interacts with mPERK and hPERK, we performed molecular docking analysis of ECH with mouse PERK (mPERK, mouse EIF2AK3, PDB ID: 3QD2) by using Glide of Schrödinger molecular modeling suite (version 2015-2). Given that the binding free energy reflects the stability and dynamic properties of intermolecular binding, we first assessed the free energy of ECH combined with mPERK with Glide of Schrödinger molecular modeling suite. The free energy of ECH combined with mPERK was −7.950 J/mol, which was low enough to form a stable complex, suggesting that ECH could be stably combined with mPERK.

To further investigate the molecular binding mode between ECH and mPERK, we performed protein-ligand interaction analysis in Glide XP visualizer to determine the potential conformation of ECH on mPERK docking and the interactions of ECH with mPERK. As shown in Figure 9C, ECH completely entered the binding-site pocket of the mPERK molecule, which was a hydrophobic cavity consisting of hydrophobic amino acids, such as phenylalanine (PHE942), glycine (GLY596), and valine (VAL603). This finding indicated that the hydrophobic property was one of the main forces governing the interaction between ECH and mPERK. There were also polar residues, such as asparagine (ASN-940) and cysteine (CYS889), in the cavity, which stabilized the ECH-mPERK complex by electrostatic interaction. Furthermore, aspartic acid (ASP896, ASP953), asparagine (ASN893), and glycine (GLY952) formed 4 hydrogen bonds with ECH. The hydrogen bonding and electrostatic interactions acted as “anchors” that stabilized the position of ECH in the mPERK binding pocket in three dimensions (Figures 9C,D).

The mPERK protein has high homology with human PERK (hPERK, human EIF2AK3, PDB ID: 4X7J), so we speculated that ECH may also bind to hPERK and have the potential for clinical AD treatment. As expected, the binding free energy of ECH combining with hPERK was −9.369 J/mol, indicating that ECH could be stably combined with hPERK. Interestingly, the free energy of hPERK combined with ECH was lower than that of mPERK, suggesting that ECH had a stronger bonding tendency with the former.

As shown in Figure 9E, ECH is exactly located within the hPERK binding pocket. Similar to mPERK, the binding cavity of hPERK is composed of hydrophobic amino acids, such as leucine (LEU643, LEU646, and LEU933), valine (VAL636), isoleucine (ILE886), and glycine (GLY602). This observation indicated that the hydrophobic property is one of the major forces in the interaction between ECH and hPERK protein, as in the interaction between ECH and mPERK. On the other hand, the interaction between ECH and hPERK was not exclusively hydrophobic. As shown in Figures 9E,F, there were polar residues, such as asparagine (ASN-942), and several electronegative residues, including aspartic acid (ASP-955) and glutamate (GLU-639), in the hPERK binding pocket. These residues further stabilize the ECH-hPERK complex by electrostatic interaction. The amino acids glutamate (GLU639), lysine (LYS622), aspartic acid (ASP955), arginine (ARG936), and asparagine (ASN942) also formed 6 hydrogen bonds with ECH. Thus, electrostatic interaction and hydrogen bonds played important roles in stabilizing the ECH-hPERK complex.

In summary, ECH can interact with mPERK and hPERK stably and tightly. The main forces of the interaction include hydrophobic interaction, electrostatic interaction, and hydrogen bonding. Therefore, ECH has shown great therapeutic potential and prospects for treating AD in human patients.

Taken together, these findings indicated that ECH repressed ERS through targeting PERK and inhibiting PERK/eIF2α activation, thus reducing the overproduction of Aβ and modulating F-actin remodeling in 2 × Tg-AD mice.

## Discussion

Almost all the approved treatments and strategies for AD are aimed at symptom management but not at targeting the underlying neuropathology (Yaffe, 2010; Kosik, 2015; Tsoi et al., 2016). As a consequence, exploring drugs to treat the pathogenesis of AD becomes imperative for AD treatment. The accumulation of Aβ triggers ERS, which plays a key role in the pathogenesis of AD (Hetz and Mollereau, 2014; Bell et al., 2016; Hughes and Mallucci, 2018). In the present study, we report for the first time that ECH ameliorated ERS via the PERK/eIF2α pathway, reversed memory impairments along with decreased Aβ accumulation and F-actin remodeling in 2 × Tg-AD mice. We demonstrated that ECH administration reduced Aβ production by inhibiting amyloidogenic APP processing and BACE1 translation without any effect on α- or γ-secretase. The inhibitory effect of ECH on BACE1 expression resulted from its inhibiting ERS-triggered PERK/eIF2α activation, which plays a pivotal role in neurodegeneration (Rozpedek et al., 2015; Hughes and Mallucci, 2018; Ohno, 2018), and eventually, ECH improved the spatial learning and memory of 2 × Tg-AD mice in the Morris water maze test. ECH promoted the interaction between PERK and FLNA and regulated F-actin remodeling. Furthermore, we confirmed that ECH bound to both mPERK and hPERK by MST assay and molecular docking analysis. This observation suggests that ECH has the great potential to be used in patients with AD. In summary, the present study provides compelling preclinical evidence that ECH can play a therapeutic role against a distinct target of AD pathogenesis and has great potential and broad prospects for the treatment of AD.

Cognitive and memory impairment is one of the most important clinical manifestations of AD, so we first tested the effect of ECH on cognitive impairment in 2 × Tg-AD mice. In this study, chronic administration of ECH (30 mg/kg b.w) daily for 6 months significantly ameliorated impairments in spatial memory performance of 2 × Tg-AD mice. This finding is consistent with other reports that ECH can ameliorate memory impairment (Shiao et al., 2017). Furthermore, as confirmed in the visible-platform test, the effect of ECH on cognitive improvement in 2 × Tg-AD mice excluded the possibility of affecting the physical and visual capacity of 2 × Tg-AD mice.

Aβ-enriched amyloid plaques are the major histopathological hallmarks of AD. Overproduction, oligomerization, and aggregation of Aβ peptides play critical and early roles in the pathogenesis of AD (Gandy, 2005; Masters and Selkoe, 2012). The oligomeric Aβ has multiple neurotoxic effects, including oxidative stress, calcium imbalance, membrane destruction, neuronal apoptosis, and ERS, etc. in the central nervous system (CNS) (Gandy, 2005; Masters and Selkoe, 2012). Therefore, the inhibition of Aβ production and accumulation is widely considered to be a potential disease-modifying approach for treating AD (Panza et al., 2019). Based on the ECH efficacy of mnemonic improvement, we hypothesized that ECH could decrease Aβ production, thereby inhibiting its neurotoxicity. To confirm this, we assessed the amyloid plaque load in both the hippocampus and cortex among the 4 groups; both of these regions are closely related to cognition. ECH reduced the plaque load in both the hippocampus and cortex in 2 × Tg-AD mice. The spatial memory ability is mainly related to the hippocampus of the brain (Yiu et al., 2011; Moodley et al., 2015). ECH can significantly reduce Aβ accumulation in the hippocampus in 2 × Tg-AD mice, which is consistent with the efficacy of ECH in improving the spatial memory of 2 × Tg-AD mice. Subsequently, we found that 6-month ECH treatment resulted in a dramatic reduction in total Aβ, Aβ42, and Aβ40 in the brains of 2 × Tg-AD mice, suggesting that ECH has a significant effect on reducing Aβ production *in vivo*. Although Aβ42 is more prone to form amyloid plaques, ECH has not shown any significant priority for the inhibition of Aβ40 or Aβ42 production. Considering that the generation of different lengths of Aβ (Aβ40 or Aβ42) depends on the γ-secretase cleavage of APP (Okochi et al., 2013), this result suggests that the target of ECH intervention in Aβ production may not be γ-secretase. Consistent with this, further experiments showed that ECH had no significant effect on the level of the catalytic subunit PS1 or the enzyme activity of γ-secretase.

BACE1 is a key rate-limiting enzyme for the production of Aβ in APP cleavage and processing. BACE1 is abnormally highly expressed in the brain tissue of AD patients or AD transgenic animals (Yang et al., 2003; Li et al., 2004; Kim et al., 2018). To further explore the effect of ECH on APP processing, we determined the effect of ECH on BACE1 transcription, translation, enzyme activity, and enzymatic cleavage. We found that ECH reduced the protein level and enzyme activity of BACE1 by inhibiting its translation without affecting its transcriptional process. This result suggested that ECH may regulate the expression of BACE1 in a post-transcriptional manner. Protein expression of BACE1 is increased in the brains of patients with AD and transgenic AD mice (Yang et al., 2003; Li et al., 2004; O’connor et al., 2008), and there is no corresponding increase in *BACE1* gene transcription (Holsinger et al., 2002; Preece et al., 2003). As a consequence, posttranscriptionally decreasing BACE1 is considered a viable strategy for the treatment of AD (Ohno, 2006; Vassar and Kandalepas, 2011; Yan and Vassar, 2014). The present study showed that ECH could better intervene in the overexpression process of BACE1 to more effectively regulate APP processing. α-Secretase is another cleavage enzyme involved in APP processing and competes with BACE1 (Fahrenholz et al., 2000). Increasing α-secretase activity is also one of the options to inhibit Aβ production in AD treatment. We found that ECH had no significant effect on the level of the catalytic subunit ADAM10 or the enzyme activity of α-secretase. Increasing target activity is often less effective than inhibiting that of a certain target for disease treatment. APP has a variety of normal physiological functions, so inhibiting its synthesis will cause certain side effects (Dawkins and Small, 2014; Montagna et al., 2019). In the present study, no significant difference in the expression of the full-length APP was detected between ECH- and vehicle-treated 2 × Tg-AD mice, indicating that ECH did not decrease Aβ production by inhibiting the synthesis of APP. These findings exhibit one of the outstanding advantages of ECH as a treatment for AD.

**FIGURE 10:**
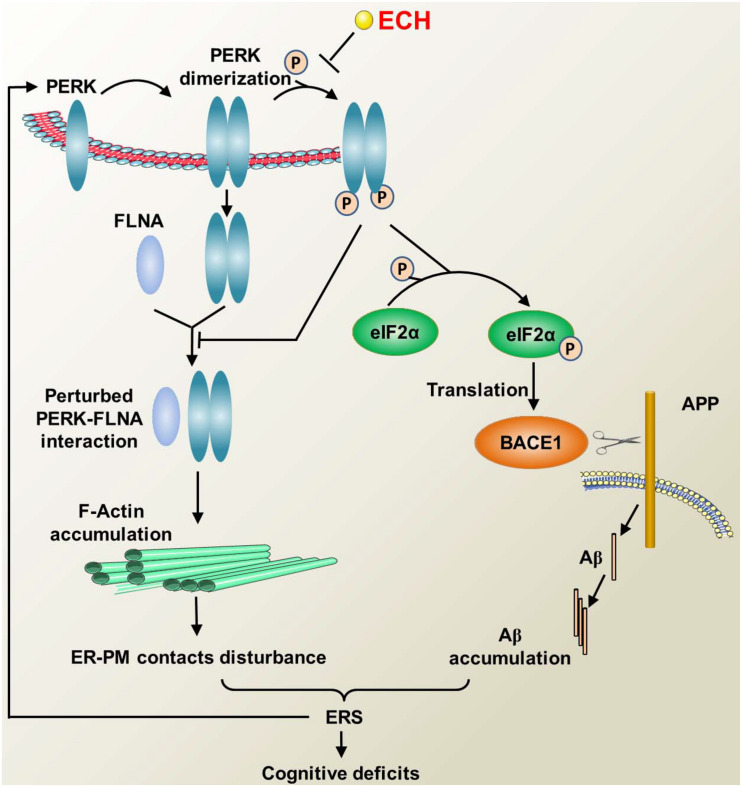
Diagram illustrating the mechanism of ECH regulating F-actin remodeling and decreasing Aβ accumulation by depressing PERK phosphorylation. PERK phosphorylation inhibits the combination of PERK dimer and FLAN, thus accelerates F-actin accumulation, then interferes with the formation of ER-PM contacts. The perturbed ER-PM contacts further deteriorate ERS. During the ERS, PERK is activated by phosphorylation, which in turn activates eIF2α, resulting in the upregulation of BACE1 translation and increased production of Aβ, and excessive accumulation of Aβ leads to ERS. The above process forms a vicious cycle that causes cognitive impairment. ECH can prevent the phosphorylation of PERK and the subsequent F-actin accumulation and Aβ overproduction, thus blocking the vicious cycle and ameliorating the cognitive impairments in AD.

Abnormal accumulation of unfolded or misfolded protein in the ER results in a disturbance in its internal environment, leading to ERS, activation of the UPR, and a series of signaling pathways associated therewith (Ron and Walter, 2007; Bell et al., 2016). The accumulation of Aβ, the main component of the important hallmark of AD, could trigger ERS and induce the UPR, which are early critical events in the course of AD (Abisambra et al., 2013; Ma et al., 2013; Devi and Ohno, 2014). Consistent with these results, we found obvious ER morphological abnormality and a marked increase in GRP78, an earlier ERS marker, in the brain of 2 × Tg-AD mice. ECH restored ER morphological abnormalities and decreased GRP78, suggesting it may rescue the ER homeostasis and thereby suppress the ERS. Dysregulated PERK/eIF2α signaling during the UPR is a common underlying mechanism of neurodegenerative diseases, including AD, PD, Huntington’s disease (HD), amyotrophic lateral sclerosis, etc. (Hetz and Mollereau, 2014; Bell et al., 2016; Ohno, 2018). PERK-dependent phosphorylation of eIF2α during the UPR is a protective mechanism that is capable of restoring protein homeostasis by rapid attenuation of further protein synthesis (Rozpedek et al., 2015; Hughes and Mallucci, 2018). However, in the case of chronic disease or severe stress conditions, such as AD, sustained phosphorylation of PERK and eIF2α can result in prolonged repression of global protein synthesis and persistent upregulation of BACE1 (Bell et al., 2016; Hughes and Mallucci, 2018). Meanwhile, the continuous upregulation of BACE1 further increases Aβ accumulation, leading to a more severe ERS, thus forming a vicious cycle that further contributes to neurodegenerative disorders and cognitive impairments (Figure 10). To elucidate the mechanisms by which ECH represses ERS and BACE1 translation, we assessed the effects of ECH on the activation of PERK and eIF2α. Consistent with many reports (Ma et al., 2013; Ma and Klann, 2014; Yang et al., 2016), our study showed that PERK and eIF2α were highly phosphorylated in the brains of 2 × Tg-AD mice, and long-term treatment of ECH dramatically decreased the phosphorylation of PERK and eIF2α, indicating that ECH inhibits ERS and the UPR by blocking PERK/eIF2α activation *in vivo*.

Upon ERS, PERK dimerized and combined with FLNA. Following the depletion of ER Ca^2+^ resulted from ERS, PERK-FLNA conjugate promotes the expansion of ER-PM juxtapositions by regulating F-actin remodeling and relocation (Van Vliet et al., 2017). ER-PM contact could refill ER-luminal Ca^2+^ and restore the balance of the cytosol and ER Ca^2+^, which is essential for restoring ER internal environment homeostasis and ameliorating ERS damages (Verfaillie et al., 2012; Van Vliet et al., 2017). Aβ induced sustained persistent ERS disturbs the interaction of PERK and FLNA. Aβ_1__–__42_ treated cells displayed obvious destruction of F-actin network and peripheral accumulation of F-actin, which in turn destroyed the formation of ER-PM contact. Given the role of ER-PM contact in ameliorating ERS injury, its reduction will further aggravate ERS, thus forming another vicious circle. Our data showed that ECH could regulate F-actin remodeling by inhibiting PERK phosphorylation, reverse the deterioration of ER-PM contact induced by Aβ, thus block the vicious circle mentioned above.

As revealed by many recent findings and the present study, the dysregulation of PERK/eIF2α is not only a detrimental downstream reaction caused by Aβ accumulation but also an important initial cause of the pathogenesis of AD (Ohno, 2014, 2018; Rozpedek et al., 2015; Bell et al., 2016; Hughes and Mallucci, 2018). As a consequence, PERK is a key hub capable of blocking the above vicious cycle (Ma and Klann, 2014; Rozpedek et al., 2015; Hughes and Mallucci, 2018; Ohno, 2018). First, dimerized PERK induced by ERS combined with FLNA. PERK-FLNA interaction promotes the formation of ER-PM contact, which in turn could recover ER inner homeostasis. Second, PERK can decrease the aberrant accumulation of Aβ and thereby suppress its neurotoxic damage. At last, PERK can repress sustained ERS and ameliorate ERS-induced neuronal impairment. In the present study, we found that ECH exerted an anti-AD effect in a “one stone/two birds” manner by targeting PERK. As shown in Figure 10, first, ECH significantly inhibited ERS by repressing the excessive and sustained activation of PERK/eIF2α, reduced the correlated ER structural abnormalities, and then ameliorated ERS-triggered neuronal degeneration. Second, ECH promoted F-actin remodeling and ER-PM contact formation by depressing the phosphorylation of PERK. Third, ECH decreased the accumulation of Aβ and the amyloid plaque load by inhibiting the translation of BACE1.

PERK is activated by autophosphorylation through dimerization (Devi and Ohno, 2014; Hughes and Mallucci, 2018). The above findings suggested that PERK may be the therapeutic target of ECH. What interested us was whether ECH could bind to mPERK. If so, we further speculated that ECH could bind hPERK and that ECH could be applied to human AD treatment. Consistent with the pharmacological efficacy of ECH shown in the present study, the MST assay and molecular docking analysis revealed that ECH stably bound both mPERK and hPERK, and the binding force included hydrophobic action, electrostatic action, and hydrogen bonding. Since the free energy of hPERK binding to ECH was lower than that of mPERK, ECH was more prone to combine with hPERK and exhibited great potential for human AD therapy.

## Data Availability Statement

The original contributions presented in the study are included in the article/supplementary material, further inquiries can be directed to the corresponding author.

## Ethics Statement

The animal study was reviewed and approved by Animal Care and Welfare Committee of Dongfang Hospital, Beijing University of Chinese Medicine, China.

## Author Contributions

TM designed the research and projected the experimental approach. YD, GH, TM, and SX performed the research and analyzed the data. YY and CZ performed the molecular docking analysis. YD, GH, and TM wrote the manuscript. All the authors contributed to the article and approved the final manuscript.

## Conflict of Interest

The authors declare that the research was conducted in the absence of any commercial or financial relationships that could be construed as a potential conflict of interest.
